# Follicle-stimulating hormone receptor expression in advanced atherosclerotic plaques

**DOI:** 10.1038/s41598-024-60962-2

**Published:** 2024-05-03

**Authors:** Nicolae Ghinea, Elisa Anamaria Liehn, Jochen Grommes, Diane Dalila Delattre, Tine Kold Olesen

**Affiliations:** 1 Département Recherche Translationnelle, Centre de Recherche, Institut Curie, 26 rue d’Ulm, 75005 Paris, France; 2 FSHR Theranostics SAS, 11 Rue de Rungis, 75013 Paris, France; 3 Institute of Molecular Medicine, University of Southern Denmark, 25 J.B. Winslow Vej, 5230 Odense, Denmark; 4 National Institute of Pathology “Victor Babes”, Splaiul Independentei 99-101, 050096 Bucharest, Romania; 5 National Heart Centre Singapore, 5 Hospital Dr, Singapore, 169609 Singapore; 6 Marienhospital Aachen, Zeise 4, 52066 Aachen, Germany; 7 Ferring France S.A.S, 7 rue Jean-Baptiste Clément, 94250 Gentilly, France; 8 Biosergen AB, Fogdevreten 2, 17165 Solna, Sweden

**Keywords:** Atherosclerosis, Atherosclerotic plaque, FSH, FSHR, FSHR1 isoform, FSHR1A02 antibody, Splenic aneurysm, Cell biology, Biomarkers, Diseases, Endocrinology, Medical research

## Abstract

Experimental evidence indicates that follicle-stimulating hormone (FSH), an essential hormone for reproduction, can act directly on endothelial cells inducing atherosclerosis activation and development. However, it remains unknown whether the FSH-receptor (FSHR) is expressed in human atherosclerosis plaques. To demonstrate the FSHR presence, we used immunohistochemical and immunoelectron microscopy involving a specific monoclonal antibody FSHR1A02 that recognizes an epitope present in the FSHR-ectodomain. In all 55 patients with atherosclerotic plaques located in carotid, coronary, femoral arteries, and iliac aneurysm, FSHR was selectively expressed in arterial endothelium covering atherosclerotic plaques and endothelia lining intraplaque neovessels. Lymphatic neovessels were negative for FSHR. M1-macrophages, foam cells, and giant multinucleated cells were also FSHR-positive. FSHR was not detected in normal internal thoracic artery. Immunoelectron microscopy performed in *ApoEKO/hFSHRKI* mice with atherosclerotic plaques, after injection in vivo with mouse anti-hFSHR monoclonal antibody FSHR1A02 coupled to colloidal gold, showed FSHR presence on the luminal surface of arterial endothelial cells covering atherosclerotic plaques. Therefore, FSHR can bind, internalize, and deliver into the plaque circulating ligands to FSHR-positive cells. In conclusion, we report FSHR expression in endothelial cells, M1-macrophages, M1-derived foam cells, giant multinucleated macrophages, and osteoclasts associated with human atherosclerotic plaques.

## Introduction

The FSH-receptor (FSHR) exists as four alternatively spliced isoforms of which three are membrane glycoproteins (FSHR1, FSHR2, FSHR3), and one is a soluble binding protein (FSHR4)^1^. Only FSHR1 and FSHR3 have known biological functions. FSHR1 is a G protein-coupled receptor that acts via the cAMP signaling pathway, whereas FSHR3 is a growth factor type I receptor that acts through calcium signaling and the MAPK/ERK pathway^1,2^.

In physiological conditions FSHR1 is mainly expressed in ovarian granulosa cells and testicular Sertoli cells^3^ as well as in the female reproductive tract^4^. FSH/FSHR1 signalling pathways involve mainly G_αs_ protein coupling and activation of adenylate cyclase resulting in the production of cyclic adenosine monophosphate (cAMP). The endothelial cells (ECs) of blood vessels in ovaries, testes and female reproductive tract express FSHR1. The endothelial FSHR1 is linked to angiogenesis^5^ and in translocation of FSH across the endothelial barrier by a process of receptor-mediated transcytosis^6^. FSHR1 expression was also observed within ECs of blood vessels associated with various pathological conditions (ex., cancer^7–12^, benign prostatic hyperplasia^7^, and endometriosis^13,14^).

Experimental evidence indicates that FSH can act directly on human arterial ECs inducing atherosclerosis activation and development^15,16^, but the relationship between FSH and atherosclerosis is not clear cut^17,18^. Recently, the hypothesis has been advanced that FSHR1 may have a role in abdominal aortic aneurysm development, in humans^19^. However, it remains unknown whether FSHR1 is expressed in human atherosclerosis lesions.

Generation of highly specific antibodies directed against the extracellular domain of FSHR1^20^, has allowed us to study the expression of FSHR1 in atherosclerotic plaques located in human carotid, coronary, femoral arteries, and iliac artery aneurysm. The results reported here establish that the G-protein coupled FSHR1 is expressed in human atherosclerotic lesions, and form a solid basis for both future studies on the role of FSHR1 in atherosclerosis and novel paths in the diagnosis and the therapy of this disease.

## Results

### Expression of FSHR1 in human carotid atherosclerotic plaques

Our initial analysis was performed on paraffin sections of human atherosclerotic plaques located in carotid with the use of mouse anti-human FSHR1-A02, recently proved suitable for FSHR1 target validation in an IHC setting for paraffin embedded tissues^11,21^. The staining pattern for FSHR1 was examined on the arterial ECs covering atherosclerotic plaques, microvascular ECs, macrophages, foam cells, giant multinucleated cells, and lymphocytes associated with the atherosclerotic plaques.

### Expression of FSHR1 in ECs

**Figure 1:**
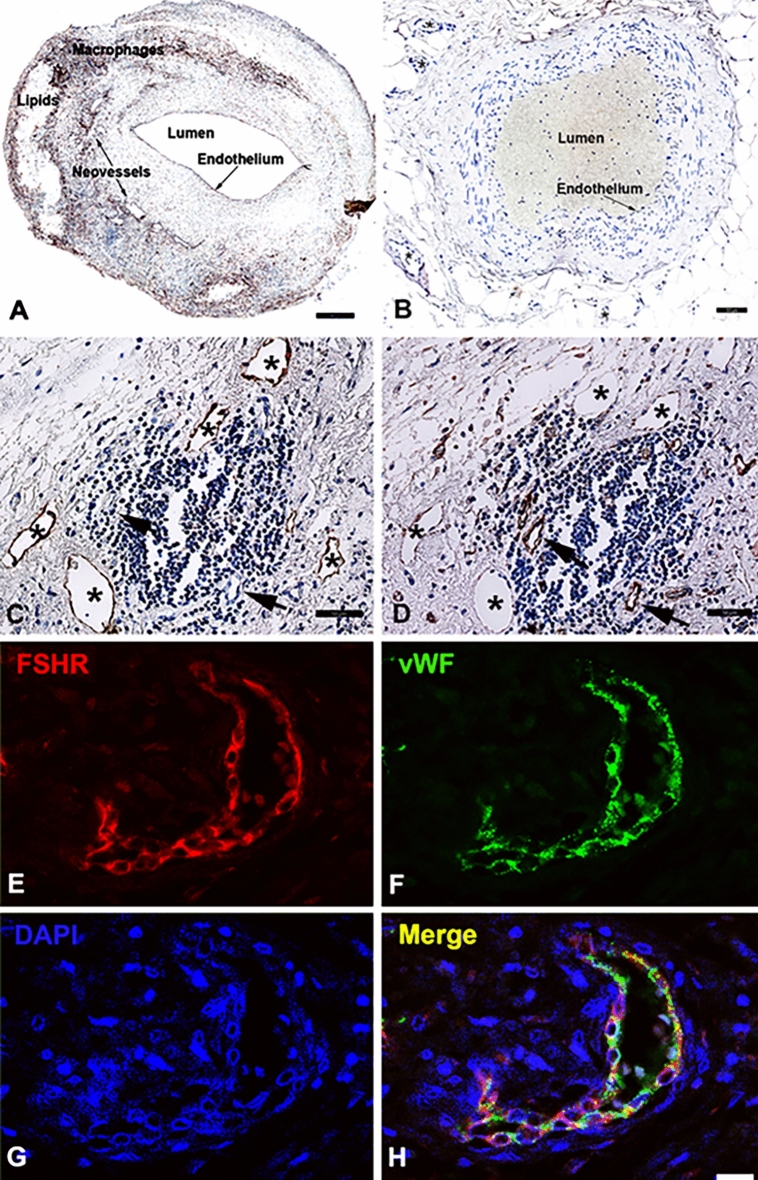
FSHR1 expression by endothelial cells in human carotid atherosclerotic plaques. A carotid atherosclerotic plaque showed strong staining of arterial and microvascular endothelial cells (Panel **A**), whereas endothelial cells of normal internal thoracic artery and its associated microvessels were negative for FSHR1 (Panel **B**). In serial paraffin sections of plaque tissue, the lymphatic neovessels stained for podoplanin with D2-40 antibody (Panel **C**) did not express FSHR1 (Panel **D**). Double immunofluorescence on carotid plaque tissue confirmed the identity of cells expressing FSHR1 (Panels **E** through **H**). An antibody against the vascular endothelial-cell marker von Willebrand factor, followed by a green-labeled secondary antibody (Panel **F**), overlapped with the signal from the mouse anti-hFSHR1A02 detected by a secondary red labeled antibody (Panel **E**). Merging of the two antibody signals with DAPI a staining fluorescent compound of nuclei (blue color) is shown in Panel H. Scale bar represents 50 µm in Panels A through D, and 25 µm in Panels E through H.

Immunohistochemical analysis of paraffin-embedded sections revealed strong staining for FSHR1 on the arterial ECs covering atherosclerotic plaques (Fig. 1A). By contrast, the arterial ECs of normal internal thoracic artery, did not express FSHR1 (Fig. 1B). The same consistent expression of FSHR1 by ECs was also detected in the endothelium lining blood microvessels formed into the plaques (Fig. 1A). The size, appearance and structure of the blood vessels’ profiles stained for FSHR1 showed that they represent capillaries (inner diameter ranges from 5 to 10 µm) and small venules (inner diameter ranges from 10 to 50 µm). The lymphatic neovessels (detected with the D2-40 antibody raised against podoplanin, a marker of lymphatics; Fig. 1C), did not express FSHR1 (Fig. 1D). FSHR1-positive ECs were identified as belonging to blood microvessels by co-staining them with an antibody against the vascular endothelial marker, von Willebrand factor (Fig. 1 panels E,F, and H). No significant differences (*p* > 0.05) were observed between the densities of FSHR1-positive blood microvessels nourishing atherosclerotic plates located in female carotid (152 ± 43) versus male carotid plaques (146 ± 53). The highest density of new FSHR1-positive blood microvessels per mm^2^ [average: 216 ± 58; median: 102 (from 41 to 265)] was noticed in atherosclerotic plaques in proximity of the inflammatory leukocyte infiltrates.

**Figure 2:**
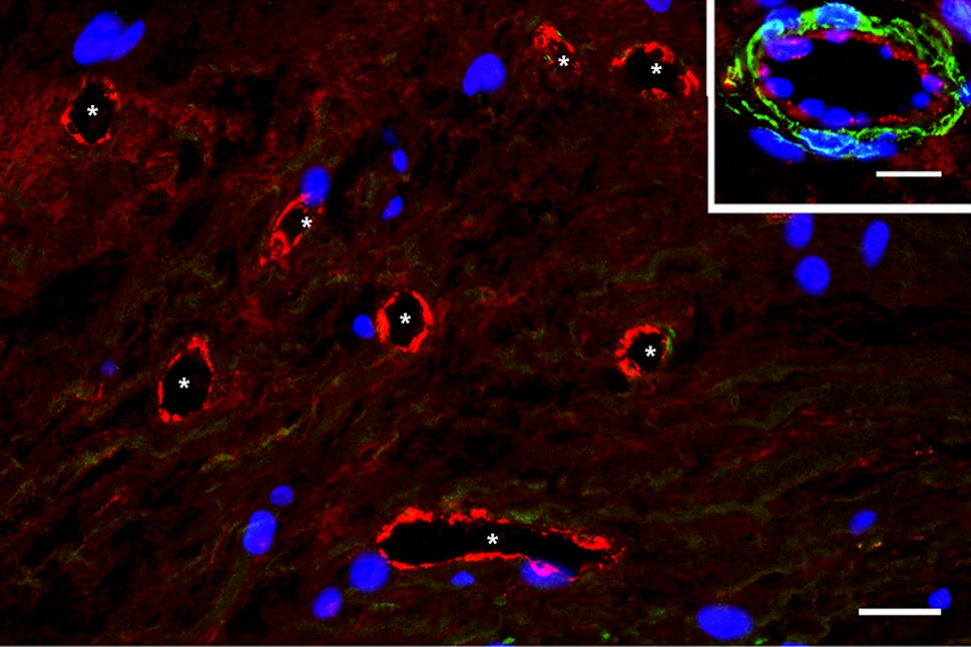
FSHR1-positive neovessels in human atherosclerotic plaques are immature blood vessels. Double immunofluorescence on atherosclerotic plaque tissue confirmed that vWF-positive blood vessels (asterisk, red staining) associated with atherosclerotic plaques lacked pericyte coverage and therefore are immature (no green staining for αSMA on mural cells). Scale bar: 50 μm. Inset: mature venule with pericyte investment (green staining). Scale bar: 50 μm.

**Figure 3:**
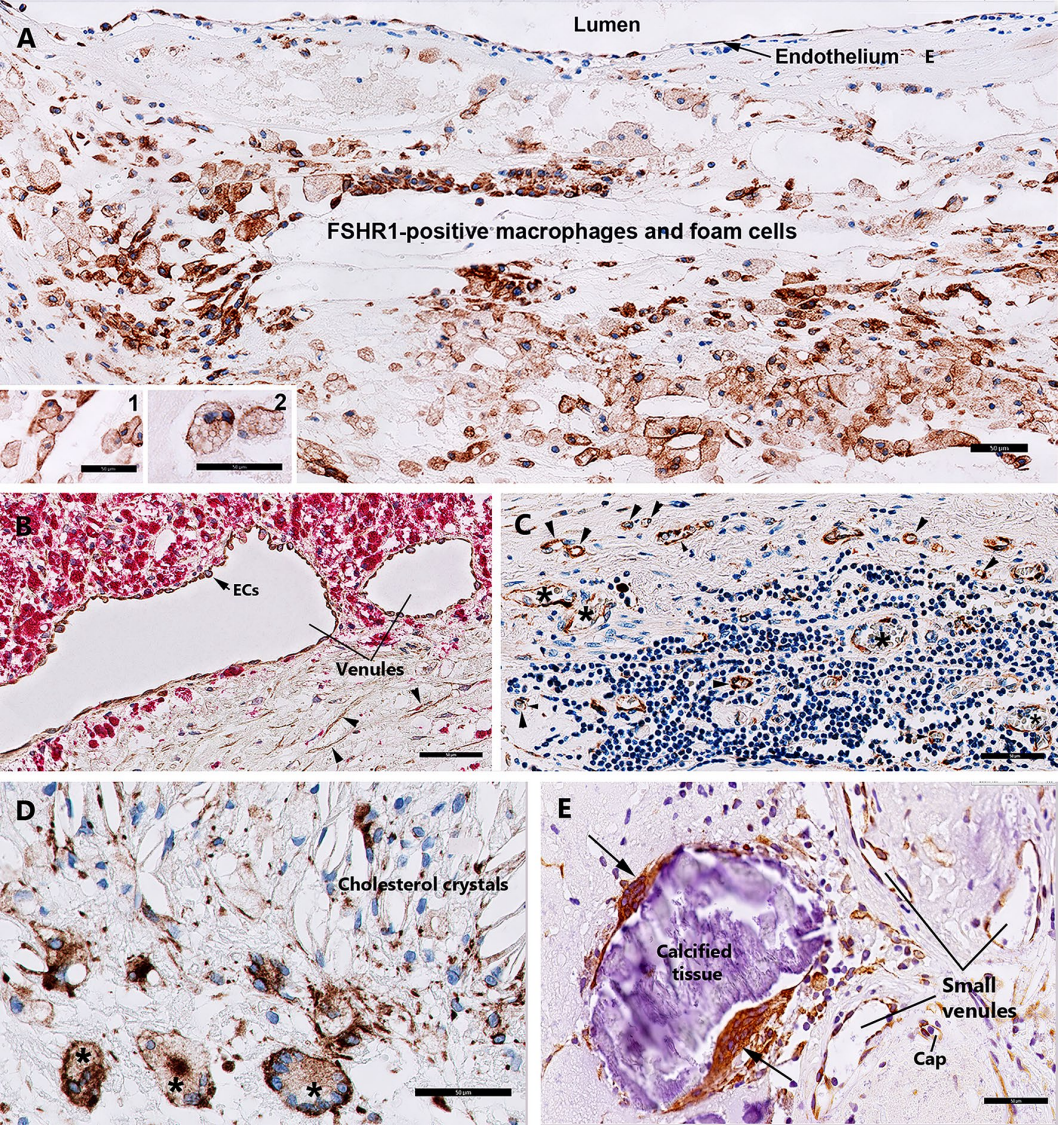
Expression of FSHR1 in other cells associated with carotid atherosclerotic plaques. While FSHR1 was detectable in arterial endothelium covering the atherosclerotic plaques (Panel **A**), endothelia lining the blood microvessels (Panels **C**, **D**), M1-macrophages (Panel **A**, inset 1), foam cells (Panel **A**, inset 2), and in giant multinucleated macrophages (Panels **D**, **E**) no staining for FSHR1 was visible in M2-macrophages expressing CD163 (Panel **B**, inset 3, red color) and lymphocytes (Panel **C**). *BV* blood vessels, *asterisks* giant multinucleated macrophages. Scale bar in all panels represents: 50 µm).

The blood microvessels we have detected in carotid atherosclerotic plaques were immature capillaries and venules (their wall consisted mainly of ECs). The ECs lining the immature blood microvessels (Fig. 2) expressed a stronger signal for von Willebrand factor than the signal associated with mature capillaries and mature venules (Fig. 3, inset). These results suggest a high concentration of Weibel Palade bodies (in which the von Willebrand factor is stored) in FSHR1-positive ECs of immature blood microvessels associated with the atherosclerotic plaques.

### Expression of FSHR1 in macrophages, foam cells, and lymphocytes

Immunohistochemical analysis performed on paraffin-embedded sections of human atherosclerotic tissues with the use of the anti-hFSHR1 monoclonal antibody A02 revealed a strong signal for FSHR1 on the cell surface membranes and in the cytoplasm of the majority of macrophages and foam cells (Fig. 3A, insets 1 and 2). Immunohistochemical analysis of colocalization of FSHR1 with CD163 (a specific marker for M2-macrophages) indicated that M2-macrophages did not express FSHR1 (Fig. 3B).

Lymphocytes, other major components of advanced atherosclerotic lesions, did not express FSHR1 either (Fig. 3C).

### Expression of FSHR1 in giant multinucleated cells

The giant multinucleated cells, formed by adhesion and fusion of adjacent macrophages are a feature of atherosclerotic plaques in the advanced stages of the disease^22^. Depending on their localisation in the carotid atherosclerotic plaques, two patterns of staining for FSHR1 were observed on giant multinucleated macrophages: (1) a strong signal for FSHR1 on the cell surface in proximity of zones rich in cholesterol crystals (Fig. 3D), and (2) a strong cytoplasmic FSHR1 staining for giant multinucleated macrophages resembling osteoclasts in proximity of calcified tissue (Fig. 3E).

### FSHR1 expression in human atherosclerotic plaques located in coronary artery, femoral artery, and in iliac artery aneurysm

Immunohistochemical studies similar to those described for human carotid atherosclerotic plaques were carried out for advanced atherosclerotic plaques located in human coronary artery (5 patients), femoral artery (5 patients), and splenic artery aneurysm (1 patient).

**Figure 4:**
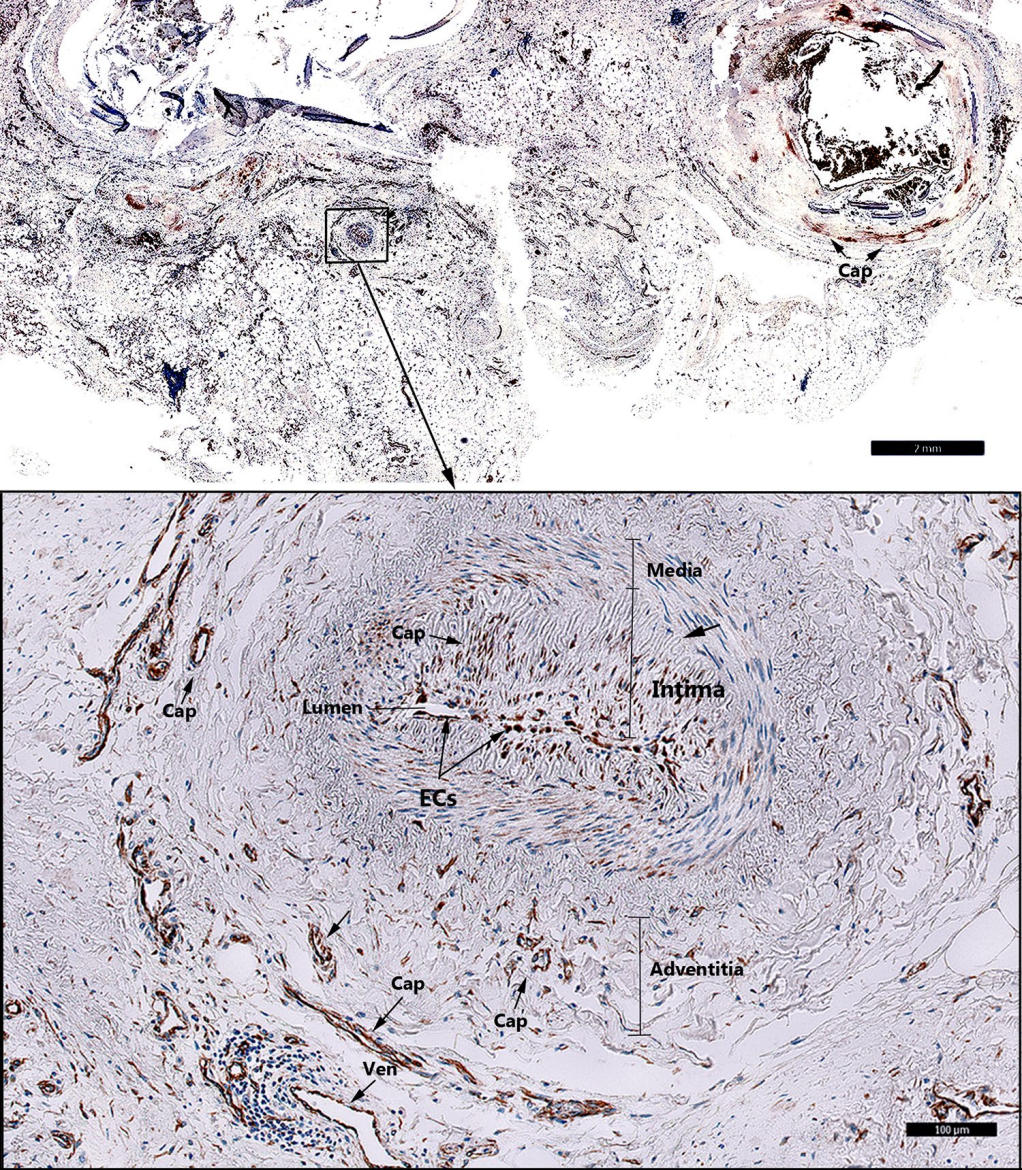
FSHR1 expression in the coronary atherosclerotic plaques. Consistent and generalized expression of endothelial FSHR1 (brown staining) in myocardium and arteries of a patient who suffered fatal myocardial infarction (Scale bar: 2 mm). Inset: Positive staining for FSHR1 in ECs associated with a small muscular artery and the myocardial microvasculature, (*BV* blood vessels, *Cap* capillary, *Ven* venule. Scale bar: 100 µm).

In coronary atherosclerotic plaques we detected consistent expression of FSHR1 by arterial ECs covering the atherosclerotic plaques, and by ECs lining capillaries and small venules (Fig. 4). The density of FSHR1-positive microvessels present in the coronary atherosclerotic plaques was 122 ± 14 blood vessels/mm^2^. Surprisingly, in one patient who suffered fatal myocardial infarction, we have noted positive staining for FSHR1 on ECs lining myocardial microvessels, small muscular arteries and their vasa vasorum (Fig. 4, detail).

**Figure 5:**
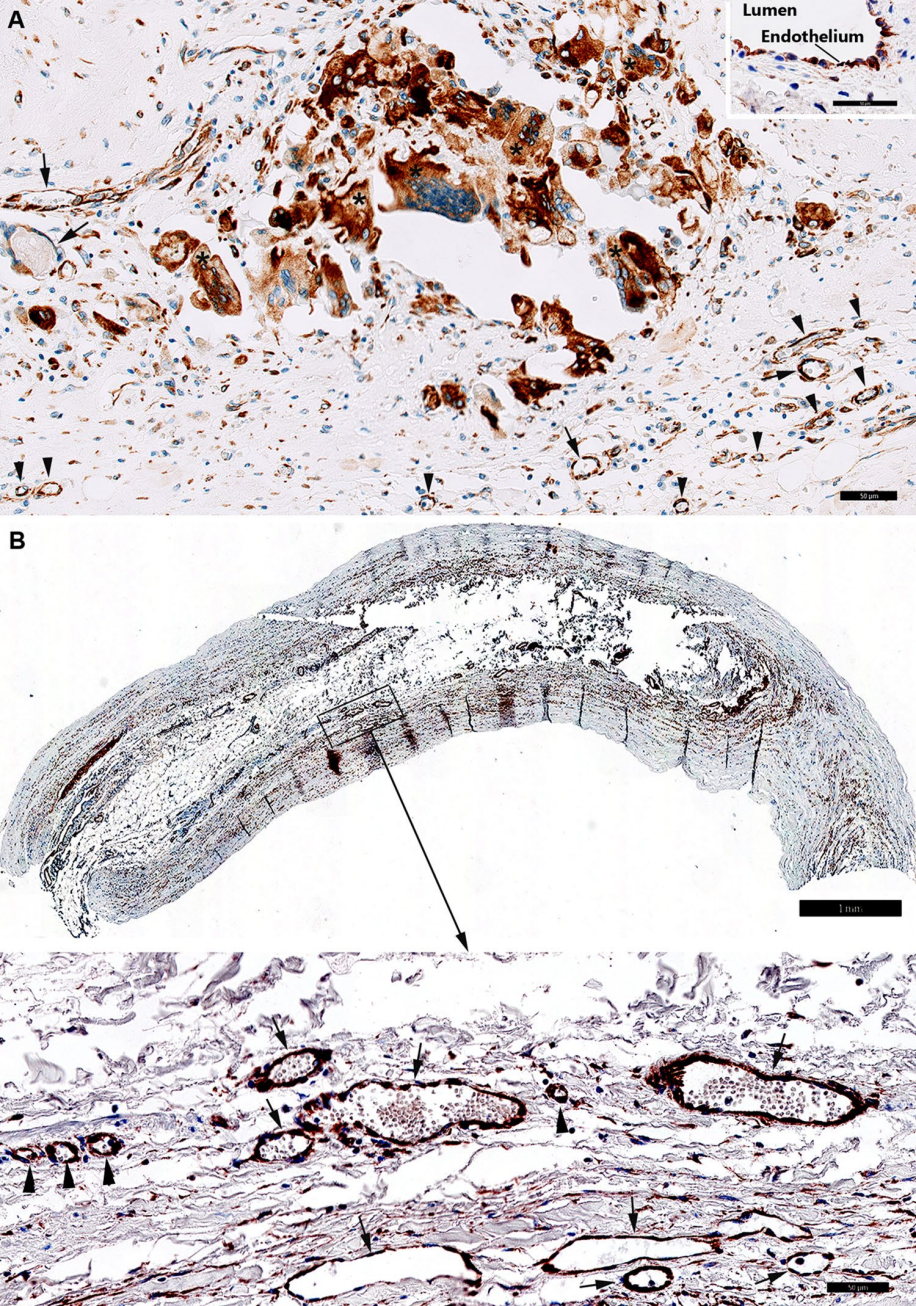
FSHR1 expression in the atherosclerotic plaques located in femoral artery and splenic artery aneurysm. (Panel **A**) Atherosclerotic plaque located in femoral artery: consistent expression of FSHR1 by endothelial cells, macrophages, and giant multinucleated macrophages (Scale bar: 50 μm). Inset: Arterial endothelial cells covering the femoral atherosclerotic plaques. (Scale bar: 50 µm). (Panel **B**) Iliac artery aneurysm (80-year old female, no smoker with no comorbidities). The aneurysmal wall examination indicates an atrophy of the muscular media caused by the atherosclerotic plaque (Scale bar: 1 mm). Inset: FSHR1-positive ECs in capillaries (arrowheads) and venules (arrows) located between the collagen fibers (Scale bar: 50 μm).

In the femoral atherosclerotic plaques (5 patients) we have detected FSHR1-positive ECs lining the luminal surface of arteries (Fig. 5A, Inset), small venules and capillaries (Fig. 5A).

The density of FSHR1-positive microvessels present in the femoral atherosclerotic plaques was 135 ± 24 microvessels/mm^2^. Strong staining for FSHR1 was also detected on the multinucleated cells resembling osteoclasts (Fig. 5A).

In the iliac artery aneurysm (1 patient, 80-year old female, no smoker with no comorbidities) we noted the atrophy of the muscular media (that atherosclerotic plaque caused) and FSHR1 expression by ECs in capillaries and venules into the plaque and between the collagen fibers (Fig. 5B).

### Endothelial FSHR1 association with cardiovascular risk factors

We used the tissue paraffin sections immunohistochemically stained for the endothelial FSHR1 expression in carotid atherosclerotic plaques of 36 patients with available data (Table 1). We analysed association of FSHR1-positive blood vessels’ density with the patients’ age and gender, hypertension, hypercholesterolemia, statin-treated patients, smoking, and diabetes.

**Table 1:** Clinical data for patients with carotid atherosclerotic plaques.

Patients’ characteristics	Patients			
	Females		Males	
	Number	Age*	Number	Age*
	12	79 (65–92)	24	72 (54–85)
Symptomatic	6 (50%)		16 (67%)	
Hypertension	7 (58%)		21 (88%)	
Hypercholesterolemia	1 (7%)		9 (38%)	
Statin-treated	2 (17%)		17 (71%)	
Diabetes	2 (17%)		5 (21%)	
Smoking	1 (7%)		9 (38%)	

* Median.

The density of vessels expressing FSHR1 in carotid lesions positively correlated with the age of the patients (r = 0.48; n = 36; *p* = 0.001).

The density of vessels expressing FSHR1 in carotid lesions was significantly higher in atherosclerotic lesions from diabetic compared to non-diabetic patients (r = 0. 71; n = 27; *p* = 2.8E−04. Table 2). There were no significant differences between the density of vessels expressing FSHR1 in atherosclerotic plaques from hypertensive vs. non-hypertensive patients, hypercholesterolemic vs. non- hypercholesterolemic, and statin-treated vs non-treated patients, and between smokers and non-smokers (Table 2).

**Table 2:** FSHR1-blood vessels’ positivity association with diabetes, smoking, hypertension, hypercholesterolemia, and statin use.

Clinical settings	Absent MVD* ± SD	Present MVD* ± SD	Correlation coefficient	No. of patients	Probability p
Diabetes	225 ± 45	406 ± 90	0.7134	27	2.8E-04
Smoking	301 ± 120	337 ± 198	0.1071	24	0.6
Hypertension	242 ± 238	340 ± 132	0.2197	19	0.32
Hypercholesterolemia	286 ± 152	363 ± 121	0.1892	19	0.4
Statin use	304 ± 151	354 ± 133	—0.3466	18	1.34

*FSHR1-positive blood vessels’ density (n/mm^2^).

### Exposure of hFSHR1 on the luminal surface of arterial endothelium covering the atherosclerotic plaques

Specific endothelial receptors could be candidates for the atherosclerotic plaque imaging and therapy because they may be directly accessible to intravenously delivered agents, which may also reach FSHR1-positive cells in the plaques after specific crossing the endothelial barrier.

**Figure 6:**
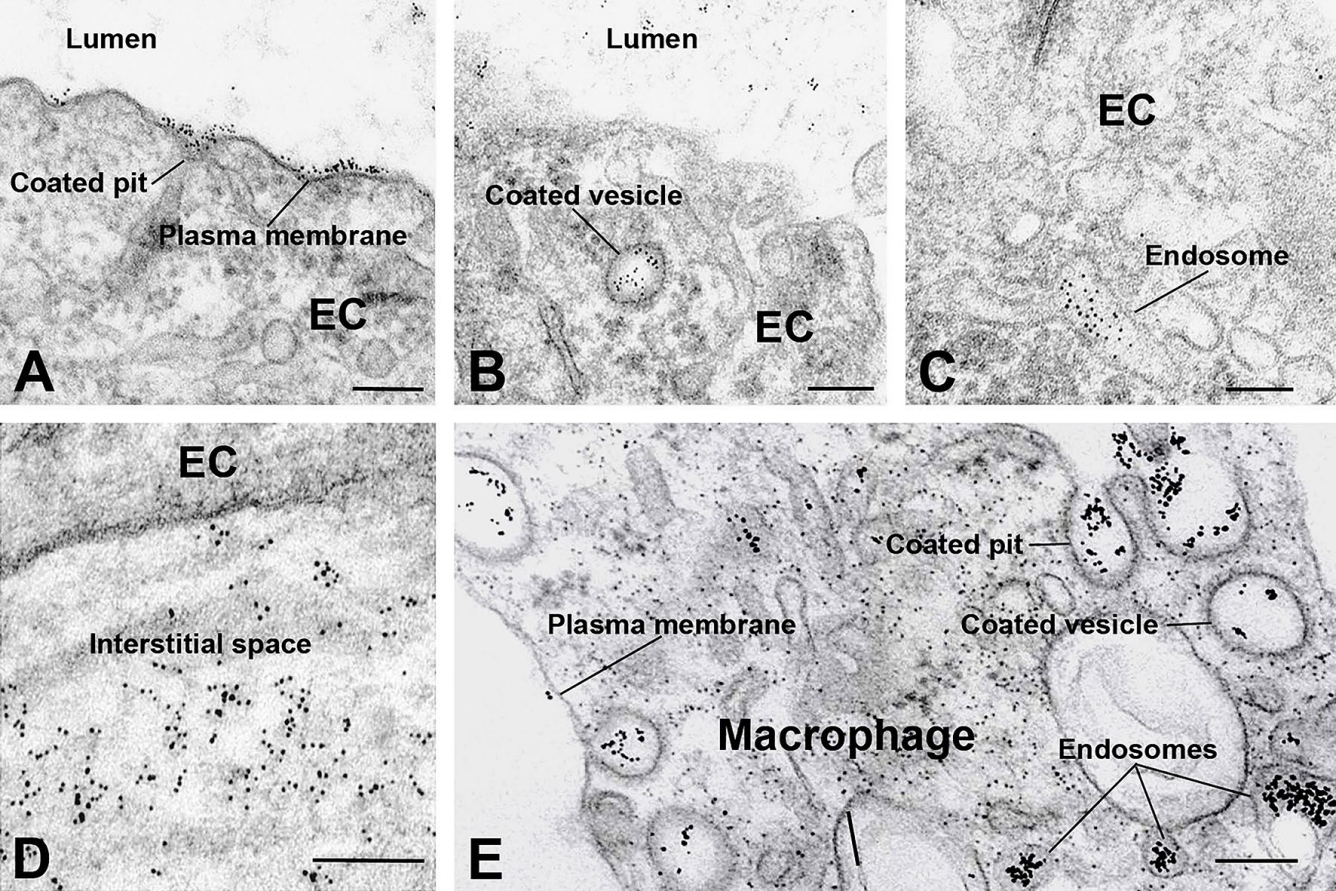
Binding and routes of transport of FSHR1-A02 antibody-colloidal gold particles by the arterial endothelial cells covering atherosclerotic plaques located in the aortic arch of *ApoEKO/hFSHR1KI* mice. (Panels **A** through **D**) After 30 min of circulation in vivo, the tracer particles (black dots) bound to the luminal plasma membrane and clathrin-coated pits were internalized via clathrin-coated vesicles in endosomes, and delivered into the subendothelial space. (Panel **E**) In contact with macrophages the FSHR1-A02 antibody-colloidal gold particles were internalized via a pathway involving clathrin-coated pits, clathrin-coated vesicles and concentrated in endosomes. (EC, endothelial cell. Scale bar: 200 nm).

Although immunohistochemical data clearly indicate that FSHR1 is expressed by various cells in atherosclerotic plaques, the question is whether the antibodies against the extracellular domain of FSHR1, when injected in vivo, have access to these FSHR1-positive cells. To answer this important question, we performed an immunoelectron study with living *ApoEKO/hFSHR1KI* mice. The atherosclerotic plaques in these mice express hFSHR1 (Supplementary Fig. 1). As a tracer, we used the mouse anti-human FSHR1A02 antibody coupled to gold particles (visible at the electron microscopic level). After 30 min of injection, the gold particles distributed on the luminal plasma membrane of endothelial cells covering the atherosclerotic plaques (Fig. 6A) were internalized by clathrin-coated pits (Fig. 6B) and clathrin-coated vesicles (Fig. 6C). A large amount of gold labeled FSHR1A02 antibody was noticed in the interstitial space of the atherosclerotic plaques (Fig. 6D) and internalized via clathrin-coated pits and clathrin-coated vesicles into the endosomes of macrophages present within the atherosclerotic plaques (Fig. 6E). This transendothelial transport of the mouse anti-human FSHR1A02 monoclonal antibody through the endothelium covering atherosclerotic plaques is analogous to the receptor-mediated transfer of circulating gonadotropin hormones hCG^23^ et FSH^6^ in testis.

## Discussion

In this study we report evidence that FSHR1, absent in normal arterial tissue, is expressed in the atherosclerotic plaques located in carotid, coronary, femoral arteries, as well as in iliac artery aneurysm affected by atherosclerosis. We also report the presence of FSHR1 on arterial endothelial cells covering the advanced atherosclerotic plaques as well as on endothelia lining microvessels present in the diseased arterial wall.

Blood microvessels in advanced vulnerable atherosclerotic plaques are known to be immature, with large gaps between endothelial cells and, therefore, with a compromised endothelial barrier that facilitates accumulation of platelets, immune cells, erythrocytes, and plasma macromolecules in the atherosclerotic plaques^24,25^. Our present immunohistochemical data indicate a high concentration of Weibel Palade bodies in endothelial cells of immature FSHR1-positive blood vessels present in the atherosclerotic plaques. Schillemans et al. demonstrated that endothelial Weibel Palade bodies’ exocytosis can be induced by cyclic AMP and Ca^2+^ elevating agonists^25^. FSH could be such an agonist because in FSHR1 expressing cells, FSH/FSHR1 signalling activates two G-proteins: Gαs (at low concentration of hormone) and G_αq_ (at high concentration) leading to cyclic AMP and Ca^2+^ production, respectively^26,27^.

Two main types of macrophages are known to be present in atherosclerotic plaques: M1-macrophages and M2-macrophages.

M1-macrophages are associated with inflammation and plaque destabilization. They produce pro-inflammatory cytokines like TNF-α and IL-6, and contribute to foam cell formation by engulfing oxidized LDL (low-density lipoprotein) particles. M2-macrophages are predominant in the later stages of atherosclerosis are considered anti-inflammatory, and therefore, supposed to stabilize the atherosclerotic plaques^28,29^. However, recent studies^30,31^ show M2-macrophages associated with vulnerable plaques located in the human carotid, which suggests that the macrophage scavenger receptor CD163 could contribute to clinical events. A strong signal for FSHR1 was detected on the pro-inflammatory M1-macrophages, foam cells, osteoclasts, and multinucleated giant cells derived from M1-macrophages in the atherosclerotic plaques for all patients we have analysed.

At present, the molecular mechanism involved in the expression of FSHR1 in the atherosclerotic lesions is unknown. Our hypothesis is that FSHR1 expression, particularly in the endothelial cells and M1-macrophages, two dominant cell types in the atherosclerotic plaques, could be induced by the oxidative stress generated by the accumulation of lipoproteins in the intima of large and medium arteries. FSHR1 expression in endothelial cells undergoing inflammatory activation should precede the initial monocyte recruitment via VCAM1, a cell adhesion molecule known to be upregulated by FSH via FSHR/Gαs/cAMP/PKA and PI3K/Akt/m TOR/NF-kB pathway^15,16^. If this mechanism is confirmed by future studies, the use of humanized monoclonal anti-hFSH should block the binding of FSH to FSHR1 and thus the deleterious role of FSH in the development of atherosclerotic plaques.

Our immunoelectron microscopy data indicate that anti-human FSHR1A02 coupled to gold particles binds specifically on the luminal surface of the arterial endothelium covering the atherosclerotic plaques. Many of the tracer particles delivered into the subendothelial space reached the FSHR1-positive macrophages and foam cells present in the atherosclerotic plaques. These results indicate that intravenously administrated anti-hFSHR1 antibodies can detect atherosclerotic plaques.

This study has focused on late stages of atherosclerotic plaques development in diseased carotid, coronary, femoral, and iliac arteries where the microenvironment created by resident cells including macrophages, foam cells, and giant multinucleated macrophages facilitates local neovascularization. The presence of FSHR1 on the surface of arterial ECs, microvascular ECs, M1-macrophages, foam cells, osteoclasts, and multinucleated giant cells makes FSHR1 an important target for both imaging and therapy of atherosclerotic lesions.

Additionally, in early atherosclerotic lesions of human carotid arteries, Jeziorska and Woolley noticed accumulation of macrophages in type I, II and III, presence of microvessels in type II and III, and absence of microvessels in type I^30^. Should FSHR1 presence also be proven in these early lesions, then humanized monoclonal anti-hFSHR1 extracellular domain antibodies coupled to fluorescent compounds combined with tomographic reconstruction would offer a longitudinal opportunity for diagnosis and treatment of atherosclerosis.

In conclusion, we report FSHR1 expression in endothelial cells, M1-macrophages, foam cells, osteoclasts, and giant multinucleated macrophages associated with human atherosclerotic plaques.

## Materials and methods

### Patients and tissue specimens

Paraffin sections for human atherosclerotic plaques (55 patients, Table 1), iliac artery aneurysm (1 patient), and normal arterial tissues (9 donors) were obtained from the Biorepository of the Department of Vascular Surgery, University Hospital Aachen, Germany. Indications for operation are peripheral arterial disease with claudication, pain, ulcer or gangrene. Operation of carotid artery stenosis is indicated if the stenosis is symptomatic and more than 50% or in case of asymptomatic stenosis more than 80% stenosis. All patients and donors have given written informed consent to the operation and the use of samples for research. The ethical approval was given by the ethic committee of the RWTH Aachen with the numbers EK181-20 and EK287/11. We confirm that all methods were performed in accordance with the relevant guidelines and regulation.

### Animals and diet

For this study we have generated *ApoEKO* mice expressing hFSHR1 by breeding *ApoEKO* female mice purchased from Jackson Laboratory (Chicago, USA; Ref: B6.129P2) with *hFSHR1KI* mice produced by CHIPHE Laboratory (Marseille, France, Ref: B6-Fshr^Tm1Ciphe^). By using a genotyping protocol (see Supporting information), 16 double homozygous *ApoEKO/hFSHR1KI* mice (8-weeks aged) were selected to induce atherosclerotic lesions with high fed cholesterol diet (Diet C1061, Altromin, Lage, Germany), and 16 double homozygous *ApoEKO/hFSHR1KI* mice were fed a standard diet for mice (Standard diet 1324, Altromin, Germany) for 22 weeks. All experiments adhere to the ARRIVE Guidelines for reporting animal research, were made in accordance with the current European regulations and approved by responsible authorities (Ministère de l’Enseignement supérieur, de la recherche et de l’innovation; Authorization number: APAFIS#24335-2020092312094134v2).

### Immunohistochemistry

We investigated by immunohistochemistry the presence of the FSHR1 in human atherosclerotic plaques associated with carotid artery (44 patients), femoral artery (5 patients), coronary arteries (5 patients), and splenic aneurysm (1 patient). Paraffin sections (5 paraffin section/patient; 5 µm thick) of human atherosclerotic lesions were immunolabeled with antibodies directed against FSHR1, the endothelial markers CD34 and von Willebrand factor, macrophage markers CD68 and CD163 (Table 3). The mouse anti-human FSHR1A02 antibody^20^ used in this study is a variant of FSHR323 antibody^3^ recently proved suitable for FSHR1 target validation in an IHC setting for paraffin embedded tissues^21^. An irrelevant mouse IgG_2a_ monoclonal antibody (MABC004, clone GC270; Sigma-Aldrich) of the same isotype as FSHR1A02 was used as control. Immunohistochemistry was carried out using an automated immunohistochemical stainer according to the manufacturer’s guidelines (Leica Bond RX, Leica Biosystems). Antigen retrieval was conducted by treatment with high temperature at pH 6 or pH 9 (Table 2).

**Table 3:** Antibodies used in this immunohistochemical study.

Antibody	Specificity	Description	Isotype	Antigen retrieval	Dilution	Incubation (min)	Supplier
FSHR1-A02	Human FSH-R1	Mouse monoclonal	IgG2a	Citrate buffer pH 6	0.2 µg/ml	30	FSHR Theranostics
F3520	Human vWF	Rabbit polyclonal		Citrate buffer pH 6	1 to 3000	30	Sigma
M7165 Qbend	CD34	Mouse monoclonal	IgG1	Tris EDTA pH 9	1 to 200	30	Dako
M3619, clone D2-40	Podoplanin	Mouse monoclonal	IgG1	Citrate buffer pH 6	1 to 100	30	Dako
KP1	CD68	Mouse monoclonal	IgG1	Tris EDTA, pH 9	1 to 500	30	Dako
NCL L-163 clone 10D6	CD163	Mouse monoclonal	IgG1	Tris EDTA, pH 9	1 to 500	30	Dako
M0851 clone	Alpha SMC actin	Mouse monoclonal	IgG1	Tris EDTA, pH 9	1 to 200	30	Dako
M9144	Irrelevant antigen	Mouse monoclonal	IgG2a	Citrate buffer pH 6	0.2 µg/ml	30	Sigma

### Quantification of blood vessels expressing FSHR1

The density of FSHR1-positive blood microvessels nourishing the atherosclerotic plaques located in human carotid, coronary, femoral, and iliac arteries has been determined on digital images from whole images of sections obtained by using the Philips Digital Ultra-Fast Scanner 1.6 RA and Philips Image Management System 2.2 RA, available in Curie Institute. This was done by counting the number of FSHR1-positive blood vessels by using ImageJ, a Java-based image processing program developed at the U.S. National Institutes of Health and available on the internet.

### Statistics

Variables are presented as mean (standard deviation, SD), median (interquartile range), or percentages. Densities of FSHR1-positive blood microvessels association with the age of patients, and densities of FSHR1-positive blood microvessels association with the absence/presence of cardiovascular risks were examined with the Pearson’s correlation coefficient. The statistical significance was evaluated using the 2-tailed t-test. Significance was considered at *p* < 0.05.

### Immunofluorescence confocal microscopy

Paraffin Sects. (5 µm) of human atherosclerotic lesions located in the carotid artery were immunolabeled with antibodies directed against the endothelial marker von Willebrand factor and FSHR1. To block the nonspecific binding of antibodies, the slides were incubated 1 h at room temperature with 2% goat serum in PBS (GS-PBS). Double labelling experiments have been done with atherosclerotic plaque tissue sections incubated with a mixture of FSHRA02 antibody (dilution 0.2 µg/ml GS-PBS) and the rabbit polyclonal anti-von Willebrand factor, a specific marker of endothelial cells (Sigma; dilution 1:3000). Mature blood vessels were determined by using a mixture of mouse anti-human alpha-SMC actin monoclonal antibody and the rabbit polyclonal anti-von Willebrand factor. A mixture of goat-anti mouse IgG-Alexa 555 and goat-anti rabbit Ig-Alexa 488 (Molecular Probes; dilution 1:750) has been used as secondary antibodies. The cell nuclei were detected by incubating slides for 10 min with DAPI (Molecular Probes; dilution 1:1000 in PBS). The slides were mounted in Dako® fluorescent mounting medium containing 15 mM sodium azide and examined with a Zeiss 510 Confocal Laser Scanning Microscope. Negative controls consisted of normal internal thoracic artery tissue specimens.

### Immunohistochemical analysis of FSHR1 protein expression in the atherosclerotic plaques in mice

The experiments were carried out on 8 double homozygous *ApoEKO/hFSHR1KI* mice (8-weeks aged) divided in two groups (A, B), each group consisting of 2 males and 2 females. The mice of groups A were fed with an atherogenic diet for mice containing 0.5% cholesterol (Diet C1061, Altromin, Germany). Mice of group B were fed a standard diet for mice (Standard diet 1324, Altromin, Germany). At 30-weeks of age the mice were sacrificed by cervical dislocation, and specimens of the aortic arch were collected, fixed in 4% formaldehyde in PBS buffer, pH 7.4 (24 h, 4 °C), dehydrated in ethylic alcohol, and finally embedded in paraffin. The expression of hFSHR1 in atherosclerotic plaques was detected with the use FSHR1A02 antibody (dilution: 1 µg/ml) followed by incubation with a streptavidin-peroxidase complex. Positive control consisted of mouse testis. Negative controls consisted of normal mouse thoracic artery, lung, liver, and pancreas.

### Immunoelectron microscopy

FSHR1A02-Au_6nm_ and mouse IgG_2a_ isotype control antibody-Au_6nm_ conjugates prepared according to standard methods were used as electron opaque tracers for localization at the electron microscope level^32^.

The experiments were carried out on 8 double homozygous *ApoEKO/hFSHR1KI* mice (2 males and 2 females/group). After light anesthesia of double homozygous *ApoEKO/hFSHR1KI* mice with a mixture of 10% Imalgene + 5% Rompun in 0.9% sodium chloride, thoracotomy, and exposure of the heart, the tracers (200 µl, A_540nm_ = 1) were injected in the left ventricle of mice and maintained in circulation for 30 min. After 30 min the mice were sacrificed and the heart and aortic arch were fixed at 4 °C with a mixture of 4% paraformaldehyde + 2.5% glutaraldehyde in 0.1 M cacodylate buffer, pH 7.2. After 24 h aortic arch specimens were collected and processed for electron microscopy. Gold particles present on random cell profiles were allocated to one of the following organelles: cell surface membrane, clathrin-coated pits and vesicles, caveolae, endosomes, multivesicular bodies, and lysosomes.

### Supplementary Information

Supplementary Information.
