# Supplementary material for: Follicle-stimulating hormone receptor expression in advanced atherosclerotic plaques
Source: Sci Rep. 2024 May 3;14:10176. doi: 10.1038/s41598-024-60962-2 (PMC11068877; doi:10.1038/s41598-024-60962-2)
Supplement: Supplementary file 1 — Supplementary Information. [file 41598_2024_60962_MOESM1_ESM.pdf]

Follicle-stimulating hormone receptor expression in advanced atherosclerotic plaques

Nicolae Ghinea, Elisa Anamaria Liehn, Jochen Grommes, Diane Dalila Delattre, Tine Kold Olesen

Supplementary material

Production of a mouse model for human atherosclerosis: Transgenic ApoE-KO mice expressing the human FSHR1

For this study we have generated ApoE-KO mice expressing the human FSHR1 by breeding ApoE-KO female mice purchased from Jackson Laboratory, Chicago, USA (Ref: B6.129P2) with hFSHR1-KI male mice from CHIPHE Laboratory, Marseille, France (Ref: B6-Fshr^{Tm1Ciphe}). By using a genotyping protocol (see below), double homozygous ApoE-KO-hFSHR-KI mice have been selected to induce atherosclerotic lesions with high fed cholesterol diet.

Genotyping protocols

STRAIN NAME: **APOE**

REAGENTS AND SOLUTIONS:

- Kapa Mouse Genotyping kit (Sigma Ref: KK7352)
- Primers 30 µM (Eurofins)
- Mineral Oil (Sigma Ref: 330779-1L)
- DNA (extracted with Kapa Mouse Genotyping kit)

REACTION MIX:

MIX	Quantity / well	
Water	6.80	µl
2X Kapa Buffer	10.00	µl
APOE1	0.60	µl
APOE2	0.30	µl
APOE3	0,30	µl
DNA	2.00	µl
Final Quantity	20.00	µl

PCR PROGRAM:

3 min	95°C	
15 sec	94°C	35 cycles
15 sec	60°C	
30 sec	72°C	
5 min	72°C	

MIGRATION:

Migration on 1.5% agarose gels.

PRIMERS SEQUENCES

- APOE1 5' GCCTAGCCGAGGGAGAGCCG3'
- APOE2 5' TGTGACTTGGGAGCTCTGCAGC3'
- APOE3 5' GCCGCCCCGACTGCATCT3'

EXPECTED AMPLIFICATIONS
WT allele: 155 bp
Modified allele: 245 bp

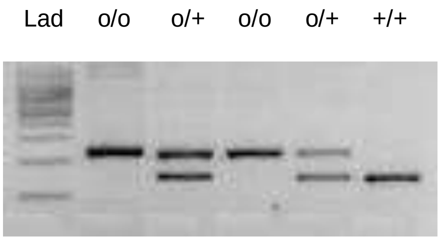

Symbols:

Lad: exact ladder 100 bp

0/0 : homozygous mApoEKO

+/+: homozygous hFSHR1-KI

0/+: heterozygous FSHR1

STRAIN NAME: **FSHR1**

REAGENTS AND SOLUTIONS:

- Kapa Mouse Genotyping kit (Sigma Ref: KK7352)
- Primers 30µM (Eurofins)
- Mineral Oil (Sigma Ref.: 330779-1L)
- DNA (extracted with Kapa Mouse Genotyping kit)

REACTION MIX:

MIX	Quantity / well
Water	6.80 µl
2X Kapa Buffer	10.00 µl
FSHRWTF	0.30 µl
FSHRKIF	0.30 µl
FSHRR	0.60 µl
DNA	20.00 µl
Final Quantity	20.00 µl

PROGRAM:

3 min	95°C	
15 sec	94°C	
15 sec	55°C	35 cycles
30 sec	72°C	
5 min	72°C	

MIGRATION: Migration on 1.5% agarose gels.

PRIMERS' SEQUENCES

- FSHRWTF 5' AAGCATCCTTTAGTGGGTCA 3'
- FSHRKIF 5' CCTTACATGTTTTACTAGCCAG 3'
- FSHRR 5' GCCAGTGATGACATCCAGAT 3'

EXPECTED AMPLIFICATIONS

WT allele: 206 bp

Modified allele: 265 bp

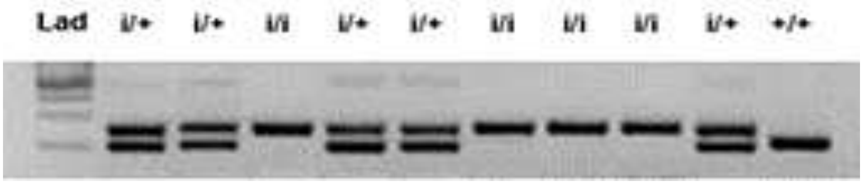

Symbols:

Lad: exact ladder 100 bp

+/+: homozygous mFSHR1

i/i : homozygous hFSHR1KI

i/+ : heterozygous FSHR1

Cholesterol contents

At age of 30 weeks 200 µl of blood / mouse was harvested and plasma has been separated from blood cells by centrifugation at 4,000g. One µl of plasma was diluted 100-fold with PBS and the total plasma cholesterol level was determined using the Abcam kit (Ref. ab65390) as indicated by supplier.

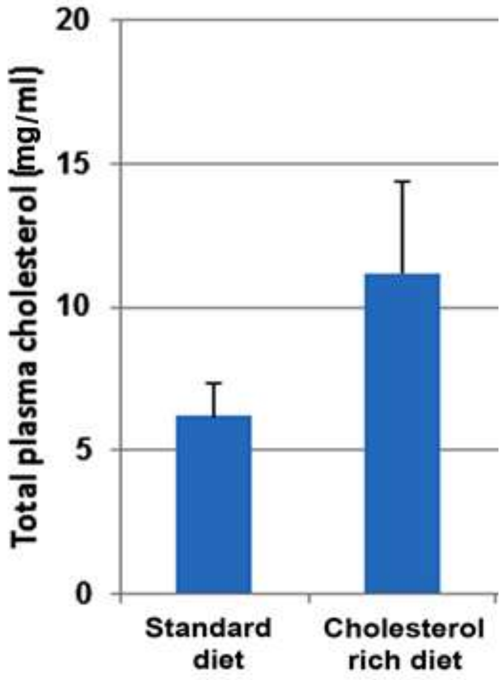

Supplementary Figure 1. Plasma cholesterol levels in double homozygous *ApoEKO / hFSHR1KI* mice. As compared with control mice fed a standard diet in which the total plasma cholesterol had an average of 6.1 ±1.1 mg/ml, in double homozygous ApoEKO / *hFSHR1KI* mice. After 20 weeks of cholesterol rich diet this value increased with 180% (10.9 ± 3.2 mg/ml plasma).

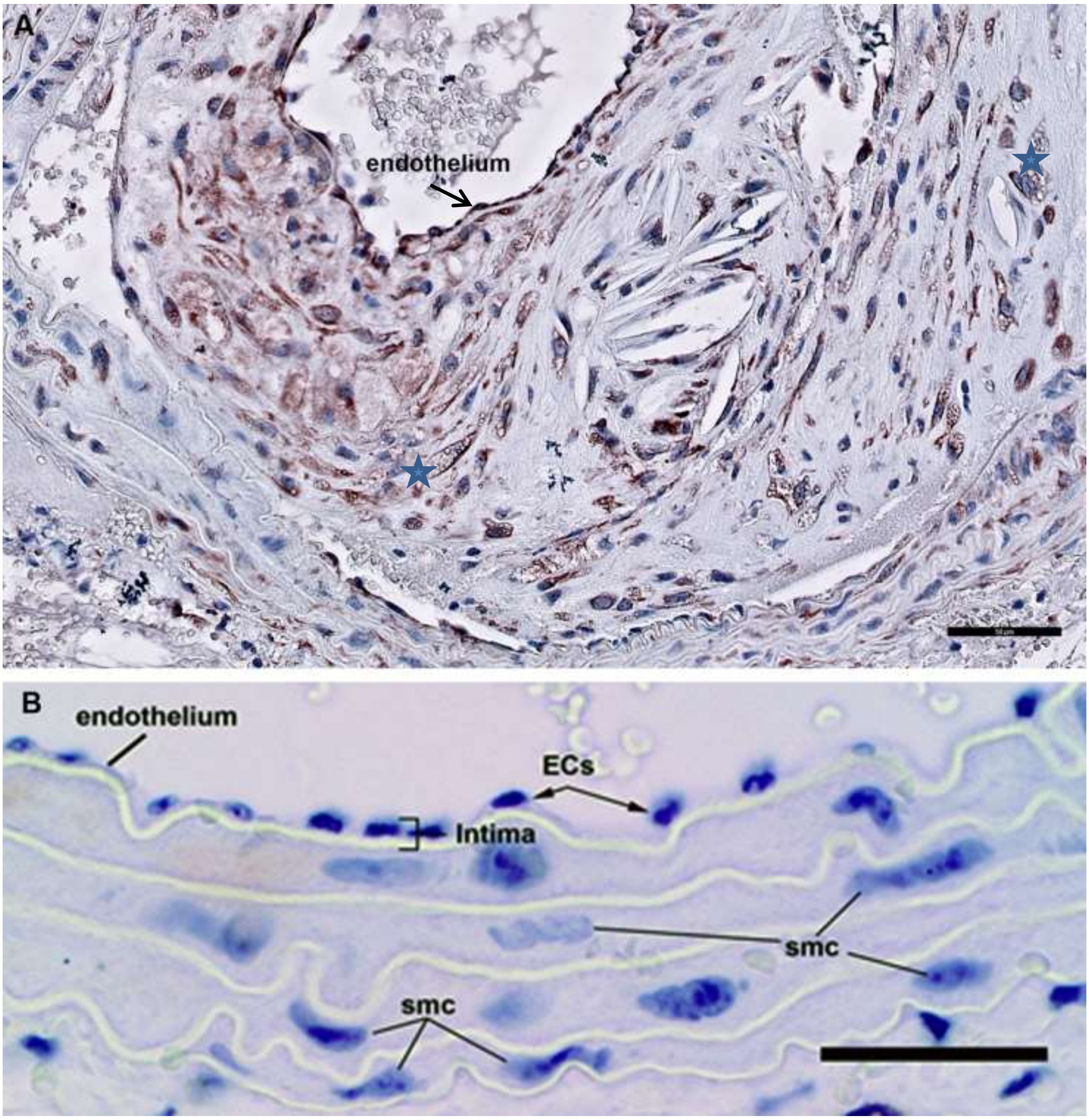

Supplementary Figure 2. FSHR1 expression in atherosclerotic plaques developed in *ApoEKO/hFSHR1KI mice* fed an atherogenic diet reach in cholesterol. (**A**) Strong signal for hFSHR1 is associated with the aortic endothelium, and foam cells (stars). No blood microvessels were seen in the plaque. (**B)** No signal was detected on normal aorta specimens from mice fed a normal standard diet. (Bar 50 µm).

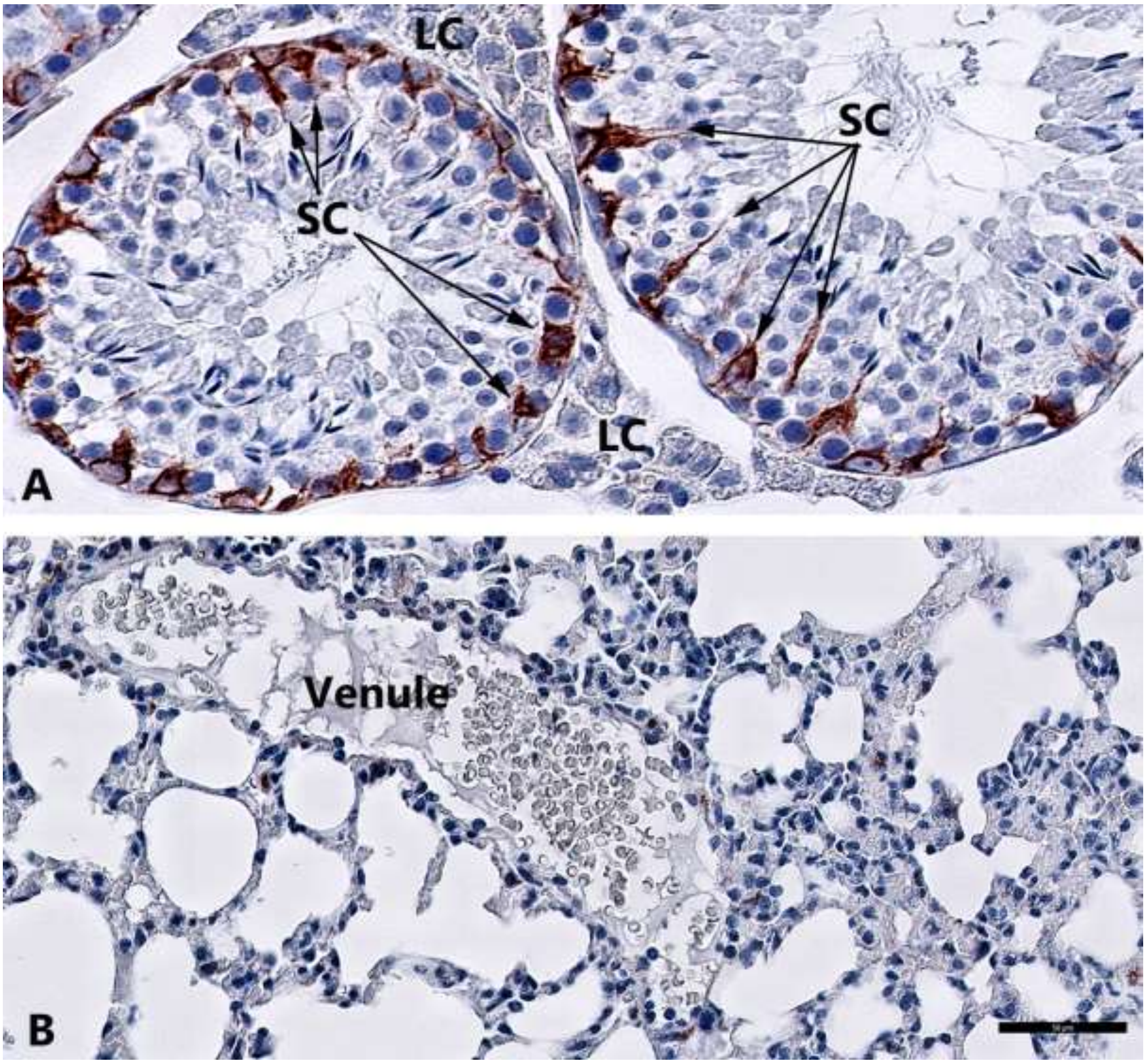

Supplementary Figure 3. Positive and negative controls for biotinylated FSHR1A02 staining.

(Panel **A**). Sertoli cells (SC) in the testes of double homozygous ApoEKO/ hFSHR1KI mice express the expected staining for hFSHR1. By contrast, Leydig cells (LC), known to express LH/hCG-receptor, are not positive for FSHR1.

Panel **B**. Normal tissue specimens from lung of double homozygous ApoEKO/ hFSHR1KI mice incubated with the biotinylated FSHRA02 did not show any hFSHR1 expression. (Bar: 50 μm).
